# Evaluation of Verigene Blood Culture Test Systems for Rapid Identification of Positive Blood Cultures

**DOI:** 10.1155/2016/1081536

**Published:** 2016-01-21

**Authors:** Jae-Seok Kim, Go-Eun Kang, Han-Sung Kim, Hyun Soo Kim, Wonkeun Song, Kyu Man Lee

**Affiliations:** Department of Laboratory Medicine, Hallym University College of Medicine, 22 Gwanpyeong-ro 170 beon-gil, Dongan-gu, Anyang-si, Gyeonggi-do 431-796, Republic of Korea

## Abstract

The performance of molecular tests using the Verigene Gram-Positive and Gram-Negative Blood Culture nucleic acid tests (BC-GP and BC-GN, resp.; Naosphere, Northbrook, IL, USA) was evaluated for the identification of microorganisms detected from blood cultures. Ninety-nine blood cultures containing Gram-positive bacteria and 150 containing Gram-negative bacteria were analyzed using the BC-GP and BC-GN assays, respectively. Blood cultures were performed using the Bactec blood culture system (BD Diagnostic Systems, Franklin Lakes, NJ, USA) and conventional identification and antibiotic-susceptibility tests were performed using a MicroScan system (Siemens, West Sacramento, CA, USA). When a single strain of bacteria was isolated from the blood culture, Verigene assays correctly identified 97.9% (94/96) of Gram-positive bacteria and 93.8% (137/146) of Gram-negative bacteria. Resistance genes* mecA* and* vanA* were correctly detected by the BC-GP assay, while the extended-spectrum *β*-lactamase CTX-M and the carbapenemase OXA resistance gene were detected from 30 cases cultures by the BC-GN assay. The BC-GP and BC-GN assays showed high agreement with conventional identification and susceptibility tests. These tests are useful for rapid identification of microorganisms and the detection of clinically important resistance genes from positive Bactec blood cultures.

## 1. Introduction

Prompt medication with suitable antibiotics has a considerable effect on mortality rates in patients suffering from bloodstream infections [1, 2]. In cases of septic shock, hours of delay in antimicrobial administration significantly increased mortality rate [3]. Therefore, of all bacterial culture tests, blood culture tests place the most emphasis on speed [4].

While conventional blood culture requires extended times for subculture, identification, and susceptibility testing, the use of molecular technologies as adjuvants during the blood culture process enables rapid identification of pathogen and antibiotic resistance, enabling patients to receive the appropriate treatment [5, 6].

The Verigene Gram-Positive Blood Culture (BC-GP) and Gram-Negative Blood Culture (BC-GN) nucleic acid tests (Nanosphere, Northbrook, IL, USA) are microarray-based assays capable of testing multiple bacterial pathogens and their antibiotic resistance simultaneously. The times of identification of microorganisms in positive blood culture broth are less than 2.5 hours with the Verigene assays. The BC-GP assay is composed of 12 bacterial targets and three resistance markers. It has been used clinically for several years in the US and many other countries, and its utility has been evaluated in multiple studies [6–9]. The BC-GN research-use-only assay was originally composed of nine bacterial targets and six resistance markers [10, 11], but the recently FDA-cleared version of BC-GN test is composed of only eight bacterial targets and six resistance markers and has become available as an* in vitro* diagnostic (IVD) test. In this study, we aimed to evaluate the performance of FDA-cleared versions of the BC-GP and BC-GN assays for the identification of Gram-positive and Gram-negative bacteria showing positive responses in the Bactec blood culture system (BD Diagnostic Systems, Franklin Lakes, NJ, USA).

## 2. Materials and Methods

### 2.1. Specimens

Two hundred and forty-nine positive blood cultures were analyzed from patients admitted to Kandgond Sacred-Heart Hospital in Seoul from March 2014 to June 2015. Duplicated blood cultures from the same patients were excluded. Gram-positive bacteria were cultured from 99 samples and Gram-negative bacteria from 150 samples. Blood specimens were inoculated into Bactec-plus aerobic/F and anaerobic/F bottles (BD Diagnostic Systems) and incubated in the Bactec Fx instrument (BD Diagnostic Systems).

### 2.2. Verigene Assays

Gram stains were performed on the samples exhibiting a positive signal for the automated blood culture system. The BC-GP and BC-GN assays were performed and the results were analyzed following manufacturer instructions. A test cartridge, utility tray, and extraction tray were loaded into the Verigene Processor Sp. 700 *μ*L of positive blood culture was added to the extraction tray sample well. Nucleic acids from positive culture were extracted and hybridized to microarray. After 2 hours, the microarray was transferred to the Verigene Reader for analysis [12].

### 2.3. Conventional Identification and Antibiotic-Susceptibility Testing

For positive blood cultures, subculturing was performed on blood-agar plates. Following overnight incubation, a pure colony was picked from the subculture for identification and susceptibility testing. The identification of Gram-positive bacteria and the susceptibility test were conducted using MicroScan Pos Combo 28, MicroScan StrepPlus Panels, and MicroScan Walkaway-96 System (Siemens, West Sacramento, CA, USA). The identification of Gram-negative bacteria and susceptibility testing were performed using the MicroScan Neg BP Combo 42 Panel (Siemens). In the event of disagreement between results from the Verizone assay and identification results using the conventional method, sequence analysis of 16S rRNA [13] and* rpoB* gene [14] was performed for Gram-positive bacteria and* Klebsiella* species, respectively.

## 3. Results

### 3.1. The BC-GP Assay

Among 99 samples from which Gram-positive bacteria were cultured, 96 showed a single strain (monomicrobial cultures) and three samples showed two strains of microorganisms (polymicrobial cultures). Among the 96 samples from which a single strain of Gram-positive bacteria was isolated, the results of 90 samples (93.8%, 95% confidence interval [CI]: 88.9%–98.6%) were concordant between the MicroScan Panel and the BC-GP assay. The BC-GP assay correctly identified the presence of* Staphylococcus aureus* (*n* = 22), except for one isolate having “no call” result (invalid result). Thirty-four* Staphylococcus epidermidis* were detected with BC-GP; however, three were identified as other coagulase-negative staphylococci (CoNS) using the MicroScan Panel. Twenty-five* Staphylococcus* spp. were detected using BC-GP, with one identified as* S. epidermidis* by the MicroScan Panel (Table 1). In the case of coagulase-negative staphylococci, for which the MicroScan Panel and the BC-GP assay showed discordant identification results in four cases, the BC-GP assay made the accurate identification, as confirmed by 16S rRNA sequencing. Enterococci were cultured in eight samples, with seven isolates, except for* Enterococcus raffinosus*, which is not included in the BC-GP assay, exhibiting concordant results between the MicroScan Panel and the BC-GP assay. The results of six streptococcal isolates were concordant between the MicroScan Panel and the BC-GP assay. The BC-GP assay accurately identified pathogens in 97.9% (94/96, 95% CI: 93.0% to 100%) of the blood culture samples from which a single Gram-positive strain was isolated (Table 1).

The BC-GP assay detected the* mecA* gene from 35 of 57 samples in which* S. aureus* or* S. epidermidis* was cultured alone, which was concordant with MicroScan susceptibility test results. From the single* Enterococcus faecium* case that exhibited vancomycin resistance, the assay detected the* vanA* gene (Table 2).

From the three samples in which two strains of microorganisms were cultured, there were differences between the MicroScan Panel and BC-GP assay results. The BC-GP assay reported* Staphylococcus* spp. with* mecA*-negative result in a sample containing methicillin-resistant* S. aureus* and* Candida albicans*. In a sample containing methicillin-susceptible* S. epidermidis* and methicillin-resistant other CoNS, the BC-GP assay reported result of* S. epidermidis* with* mecA* and* Staphylococcus* spp. (Table 3).

### 3.2. The BC-GN Assay

Among the 150 samples from which Gram-negative bacteria were cultured, 146 showed a single strain and four showed at least two strains of microorganisms. Of the 146 monomicrobial cultures, 138 (94.5%) isolates belonged to microorganisms included in the BC-GN assay (Table 4).* Escherichia coli* was most frequently isolated, with 82 samples (59.4%), followed by* Acinetobacter* spp. with 22 samples (15.9%),* Klebsiella pneumoniae* with 19 samples (13.8%),* Enterobacter* spp. with four samples (2.9%),* Proteus* spp. with four samples (2.9%),* Citrobacter* spp. with three samples (2.2%), and finally* Klebsiella oxytoca* and* Pseudomonas aeruginosa* with two samples each (1.4%). Eight samples showed microorganisms not included in the BC-GN assay, of which three samples showed* Stenotrophomonas maltophilia*, with* Aeromonas hydrophila*,* Burkholderia cepacia*, and* Morganella morganii* cultured in one sample each. From two samples,* K. pneumoniae* was detected by the MicroScan Panel but failed to be detected by the BC-GN assay. These isolates were confirmed as* Klebsiella variicola* by* rpoB* gene sequencing.

The overall concordant rates between the BC-GN assay and the MicroScan Panel for Gram-negative monomicrobial cultures were 93.8% (137/146, 95% CI: 90.0% to 97.7%), and 99.3% (137/138, 95% CI: 98.6% to 100%) showed concordant results when only the microorganisms included in the BC-GN assay were considered (Table 4). One sample, from which a single strain was subcultured and* E. coli* was later detected by the MicroScan Panel, exhibited a positive response for both* E. coli* and* K. pneumoniae* in the BC-GN assay.

Two of the four samples from which at least two strains of microorganisms were cultured showed a coculture of* E. coli* and* K. pneumoniae*, with the MicroScan Panel and the BC-GN assay exhibiting concordant results. One sample showed a coculture of* Enterobacter* spp. and* Serratia marcescens* and exhibited a positive response for* Enterobacter* spp. and* K. oxytoca* in the BC-GN assay. In another sample, the three microorganisms,* Acinetobacter* spp.,* Enterobacter* spp., and* S. marcescens*, were simultaneously cultured, with the BC-GN assays showing a positive response for* Acinetobacter* spp.,* Enterobacter* spp., and* K. oxytoca* (Table 3).

Resistance markers were detected from 30 samples with the BC-GN assay. Among the 84* E. coli* samples, 14 exhibited an extended-spectrum *β*-lactamase (ESBL) CTX-M positive response with the BC-GN assay, while* Acinetobacter* spp. exhibited a carbapenemase OXA positive response in 15 of 23 cultured samples, and* K. pneumoniae* exhibited a CTX-M positive response in one of 23 cultured samples (Table 2). All CTX-M positive isolates (*E. coli*,* K. pneumoniae*) were resistant to cephalosporins and susceptible to carbapenems, and all OXA positive isolates (*Acinetobacter* spp.) showed multidrug resistant phenotype by antibiotic-susceptibility testing (Table 5).

## 4. Discussion

Many studies have evaluated the performance of the BC-GP and BC-GN assays for the prompt detection of bacterial pathogens and resistance markers from positive blood cultures [6–12]. Most studies have only evaluated either the BC-GP assay or the BC-GN assay and have assayed simulated specimens, which were made from preserved isolates, along with fresh or frozen specimens. This study tested the performance of both the BC-GP and BC-GN assays targeting fresh clinical specimens using the Bactec blood culture system.

The BC-GP assay has been appraised for superior identification and resistance marker detection from monomicrobial-positive blood cultures [6–8]. We demonstrated that the BC-GP assay results were accurate in 97.9% of the samples in which a single strain of Gram-positive bacteria was cultured. The BC-GP assay also efficiently detected the* mecA* and* vanA* genes from samples in which a single strain of Gram-positive bacteria was cultured.

A previous study reported that one of 20* K. pneumoniae* isolates was not detected by the BC-GN assay [9], and another study reported failure to detect two of 14 isolates [11]. Recently, the BC-GN assay exhibited false-negative results most frequently for* K. variicola*, and it was speculated that it might be wrongly identified as* K. pneumoniae* during biochemical identification [12].* K. variicola* is phenotypically similar to* K. pneumoniae* and has been frequently isolated from clinical specimens since the introduction of molecular detection methods [15, 16]. The BC-GN assay failed in two samples to detect* K. pneumoniae*, which was identified by the MicroScan Panel. We identified the two isolates of* K. variicola* which were misidentified as* K. pneumoniae* by the MicroScan Panel in this study.

In one sample, although the MicroScan Panel identified* E. coli*, the BC-GN assay exhibited a positive response for both* E. coli* and* K. pneumoniae*. The analytical sensitivity of the BC-GN assay is 10^5^–10^7^ colony-forming units (CFUs), which is sensitive when compared to the count number for a positive response from a blood culture bottle (10^8^–10^9^ CFUs) [17]. It is possible that the conventional culture only isolated* E. coli* due to the insufficient number of* K. pneumoniae* present in the blood culture bottle.

The BC-GN assay has the disadvantage of not including as detection targets Gram-negative microorganisms that are frequently isolated [12]. In this study, 5.5% of Gram-negative isolates cultured as a single strain were microorganisms not included in the BC-GN assay and, therefore, were only capable of being identified with the MicroScan Panel.

The BC-GN assay was able to detect resistance markers for CTX-M and OXA from 30 samples. While earlier studies detected resistance markers from stored bacterial strains, we were able to validate the ability of the BC-GN assay to detect resistance markers from clinical specimens.

The BC-GN assay is designed to detect 6 common resistant genes, but there are other resistance genes and mechanisms. The negative results for any of resistance markers by the BC-GN assay cannot exclude a resistant isolate.

The accuracy of the BC-GP and BC-GN assays has been reported to be relatively low for the direct identification of microorganisms in polymicrobial cultures [6, 12]. The same issue exists in matrix-assisted laser desorption/ionization time-of-flight mass spectrometry or other molecular assays, such as the FilmArray blood culture identification (BioFire Diagnostics, Salt Lake City, UT) [18, 19]. Among the 249 samples in this study, seven (2.8%) exhibited polymicrobial culture results. There was disagreement in the results between the Verigene assay and the MicroScan Panel in five of the seven samples. Polymicrobial results from Verigene assays should be confirmed by conventional identification and antimicrobial susceptibility test.

In conclusion, the BC-GP and BC-GN assays demonstrated high analytical accuracy for clinical specimens and exhibited consistent results with conventional identification and sensitivity tests. Employing these tests in positive Bactec blood cultures will be useful for the rapid identification of microorganisms and the detection of clinically important resistance genes.

## Figures and Tables

**Table 1:** Identification of the Verigene Gram-Positive Blood Culture in monomicrobial samples.

Organism	Number of isolates	Number of correctly identified isolates	
		Verigene	MicroScan
*Staphylococcus*			
*S. aureus*	23	22	23
*S. epidermidis*	34^a^	34	31
*Staphylococcus *spp.	25^b^	25	24
*Streptococcus*			
*S. agalactiae*	1	1	1
*S. anginosus*	1	1	1
Other streptococci	4	4	4
*Enterococcus*			
*E. faecalis*	5	5	5
*E. faecium*	2	2	2
*E. raffinosus*	1^c^		1

Total	96	94	92

^a^Three isolates identified *Staphylococcus *spp. by MicroScan but *S. epidermidis* by 16S rRNA sequencing.

^b^An isolate identified *S. epidermidis *by MicroScan but *S. hominis* by 16S rRNA sequencing.

^c^Not included among the targets of Verigene Gram-Positive Blood Culture.

**Table 2:** Resistance markers detected by the Verigene assays.

Organism	Number of isolates	Number of resistance markers by the Verigene assays			
		*mecA*	*vanA*	CTX-M	OXA
*S. aureus*	23	8			
*S. epidermidis*	34	27			
*E. faecium.*	2		1		
*E. coli*	82			14	
*K. pneumoniae*	19			1	
*Acinetobacter* spp.	22				15

**Table 3:** Results of the Verigene assays in polymicrobial samples (*n* = 7).

Case	MicroScan result	Verigene result
1	*S. epidermidis *(MR^a^), *Candida albicans*	*Staphylococcus *spp.
2	*S. epidermidis, Staphylococcus *spp. (MR)	*mecA (+) S. epidermidis*, *Staphylococcus *spp.
3	*S. epidermidis* (MR), *Streptococcus *spp.	*mecA (+) S. epidermidis*, *Staphylococcus *spp., *Streptococcus *spp.
4	*E. coli*, *K. pneumoniae*	*E. coli*, *K. pneumoniae*
5	*E. coli*, *K. pneumoniae*	*E. coli*, *K. pneumoniae*
6	*Enterobacter *spp., *S. marcescens*	*Enterobacter *spp., *K. oxytoca*
7	*Acinetobacter* spp., *Enterobacter *spp., and *S. marcescens*	*Acinetobacter* spp., *Enterobacter* spp., and *K. oxytoca*

^a^MR, methicillin resistant.

**Table 4:** Identification of the Verigene Gram-Negative Blood Culture in monomicrobial samples.

Organism	Number of isolates	Number of correctly identified isolates	
		Verigene	MicroScan
*E. coli*	82	81	82
*K. pneumoniae*	19^a^	19	19
*Acinetobacter* spp.	22	22	22
*Enterobacter* spp.	4	4	4
*Proteus* spp.	4	4	4
*Citrobacter *spp.	3	3	3
*K. oxytoca*	2	2	2
*P. aeruginosa*	1	1	1
Not targeted organism	8^b^		6

Total	146	137	144

^a^Two isolates identified *Klebsiella pneumoniae* by MicroScan but *Klebsiella variicola* by *rpoB* sequencing.

^b^Included three *Stenotrophomonas maltophilia*, two *K. variicola,* one *Aeromonas hydrophila*, one *Burkholderia cepacia*, and one* Morganella morganii.*

**Table 5:** BC-GN resistance markers and results of antibiotic-susceptibility testing.

BC-GN test gene	Organism	Antibiotic-susceptibility test						
		CAZ	CTX	IPM	MEM	GEN	AMK	CIP
CTX-M	*E. coli*	R	R	S	S	S	S	R
	*E. coli*	R	R	S	S	S	I	R
	*E. coli*	R	R	S	S	S	S	R
	*E. coli*	R	R	S	S	R	S	R
	*E. coli*	R	R	S	S	R	S	S
	*E. coli*	R	R	S	S	S	I	R
	*E. coli*	R	R	S	S	R	S	R
	*E. coli*	R	R	S	S	R	S	R
	*E. coli*	R	R	S	S	R	S	R
	*E. coli*	R	R	S	S	S	S	S
	*E. coli*	R	R	S	S	R	S	R
	*E. coli*	R	R	S	S	R	S	R
	*E. coli*	R	R	S	S	S	S	S
	*E. coli*	R	R	S	S	R	S	R
	*K. pneumoniae*	R	R	S	S	S	S	S

OXA	*Acinetobacter* spp.	R	R	R	R	R	R	R
	*Acinetobacter* spp.	R	R	R	R	R	R	R
	*Acinetobacter* spp.	R	R	R	R	R	R	R
	*Acinetobacter* spp.	R	R	R	R	R	R	R
	*Acinetobacter* spp.	R	R	R	R	R	S	R
	*Acinetobacter* spp.	R	R	R	R	R	R	R
	*Acinetobacter* spp.	R	R	R	R	R	S	R
	*Acinetobacter* spp.	R	R	R	R	R	R	R
	*Acinetobacter* spp.	R	R	R	R	R	R	R
	*Acinetobacter* spp.	R	R	R	R	R	R	R
	*Acinetobacter* spp.	R	R	R	R	R	S	R
	*Acinetobacter* spp.	R	R	R	R	R	R	R
	*Acinetobacter* spp.	R	R	R	R	R	S	R
	*Acinetobacter* spp.	R	R	R	R	R	R	R
	*Acinetobacter* spp.	R	R	R	R	R	R	R

CAZ, ceftazidime; CTX, cefotaxime; IPM, imipenem; MEM, meropenem; GEN, gentamicin; AMK, amikacin; CIP, ciprofloxacin; R, resistant; S, susceptible; and I, intermediate.

## References

[1] Trenholme G. M., Kaplan R. L., Karakusis P. H. (1989). Clinical impact of rapid identification and susceptibility testing of bacterial blood culture isolates. *Journal of Clinical Microbiology*.

[2] Kang C.-I., Kim S.-H., Wan B. P. (2005). Bloodstream infections caused by antibiotic-resistant gram-negative bacilli: risk factors for mortality and impact of inappropriate initial antimicrobial therapy on outcome. *Antimicrobial Agents and Chemotherapy*.

[3] Kumar A., Roberts D., Wood K. E. (2006). Duration of hypotension before initiation of effective antimicrobial therapy is the critical determinant of survival in human septic shock. *Critical Care Medicine*.

[4] Moore D. F., Hamada S. S., Marso E., Martin W. J. (1981). Rapid identification and antimicrobial susceptibility testing of gram-negative bacilli from blood cultures by the automicrobic system. *Journal of Clinical Microbiology*.

[5] Bhatti M. M., Boonlayangoor S., Beavis K. G., Tesic V. (2014). Evaluation of filmarray and verigene systems for rapid identification of positive blood cultures. *Journal of Clinical Microbiology*.

[6] Samuel L. P., Tibbetts R. J., Agotesku A., Fey M., Hensley R., Meier F. A. (2013). Evaluation of a microarray-based assay for rapid identification of Gram positive organisms and resistance markers in positive blood cultures. *Journal of Clinical Microbiology*.

[7] Wojewoda C. M., Sercia L., Navas M. (2013). Evaluation of the verigene gram-positive blood culture nucleic acid test for rapid detection of bacteria and resistance determinants. *Journal of Clinical Microbiology*.

[8] Beal S. G., Ciurca J., Smith G. (2013). Evaluation of the nanosphere verigene gram-positive blood culture assay with the versaTREK blood culture system and assessment of possible impact on selected patients. *Journal of Clinical Microbiology*.

[9] Dodémont M., De Mendonça R., Nonhoff C., Roisin S., Denis O. (2015). Evaluation of verigene gram-positive blood culture assay performance for bacteremic patients. *European Journal of Clinical Microbiology and Infectious Diseases*.

[10] Mancini N., Infurnari L., Ghidoli N. (2014). Potential impact of a microarray-based nucleic acid assay for rapid detection of gram-negative bacteria and resistance markers in positive blood cultures. *Journal of Clinical Microbiology*.

[11] Dodémont M., De Mendonça R., Nonhoff C., Roisin S., Denis O. (2014). Performance of the verigene gram-negative blood culture assay for rapid detection of bacteria and resistance determinants. *Journal of Clinical Microbiology*.

[12] Ledeboer N. A., Lopansri B. K., Dhiman N. (2015). Identification of Gram-negative bacteria and genetic resistance determinants from positive blood culture broths by use of the Verigene Gram-negative blood culture multiplex microarray-based molecular assay. *Journal of Clinical Microbiology*.

[13] Kim M., Heo S. R., Choi S. H. (2008). Comparison of the MicroScan, VITEK 2, and Crystal GP with 16S rRNA sequencing and MicroSeq 500 v2.0 analysis for coagulase-negative *Staphylococci*. *BMC Microbiology*.

[14] Alves M. S., Da Silva Dias R. C., De Castro A. C. D., Riley L. W., Moreira B. M. (2006). Identification of clinical isolates of indole-positive and indole-negative *Klebsiella* spp.. *Journal of Clinical Microbiology*.

[15] Alves M. S., da Silva Dias R. C., de Castro A. C. D., Riley L. W., Moreira B. M. (2006). Identification of clinical isolates of indole-positive and indole-negative *Klebsiella* spp.. *Journal of Clinical Microbiology*.

[16] Seki M., Gotoh K., Nakamura S. (2013). Fatal sepsis caused by an unusual *Klebsiella* species that was misidentified by an automated identification system. *Journal of Medical Microbiology*.

[17] Marla S., Dado M., Gerstein C. Development of nanosphere’s verigene BC-GN test for rapid detection of gram-negative bacteria and resistance determinants directly from positive blood culture.

[18] La Scola B., Raoult D. (2009). Direct identification of bacteria in positive blood culture bottles by matrix-assisted laser desorption ionisation time-of-flight mass spectrometry. *PLoS ONE*.

[19] Altun O., Almuhayawi M., Ullberg M., Ozenci V. (2013). Clinical evaluation of the FilmArray blood culture identification panel in identification of bacteria and yeasts from positive blood culture bottles. *Journal of Clinical Microbiology*.

